# Cytoskeleton Reorganization in EndMT—The Role in Cancer and Fibrotic Diseases

**DOI:** 10.3390/ijms222111607

**Published:** 2021-10-27

**Authors:** Wojciech Michał Ciszewski, Marta Ewelina Wawro, Izabela Sacewicz-Hofman, Katarzyna Sobierajska

**Affiliations:** Department of Molecular Cell Mechanisms, Medical University of Lodz, Mazowiecka 6/8, 92215 Lodz, Poland; wojciech.ciszewski@umed.lodz.pl (W.M.C.); marta.wawro@umed.lodz.pl (M.E.W.); izabela.sacewicz-hofman@umed.lodz.pl (I.S.-H.)

**Keywords:** EndMT, cancer, fibrosis, cytoskeleton remodeling

## Abstract

Chronic inflammation promotes endothelial plasticity, leading to the development of several diseases, including fibrosis and cancer in numerous organs. The basis of those processes is a phenomenon called the endothelial–mesenchymal transition (EndMT), which results in the delamination of tightly connected endothelial cells that acquire a mesenchymal phenotype. EndMT-derived cells, known as the myofibroblasts or cancer-associated fibroblasts (CAFs), are characterized by the loss of cell–cell junctions, loss of endothelial markers, and gain in mesenchymal ones. As a result, the endothelium ceases its primary ability to maintain patent and functional capillaries and induce new blood vessels. At the same time, it acquires the migration and invasion potential typical of mesenchymal cells. The observed modulation of cell shape, increasedcell movement, and invasion abilities are connected with cytoskeleton reorganization. This paper focuses on the review of current knowledge about the molecular pathways involved in the modulation of each cytoskeleton element (microfilaments, microtubule, and intermediate filaments) during EndMT and their role as the potential targets for cancer and fibrosis treatment.

## 1. Introduction

The vascular endothelium is composed of a monolayer of tightly connected endothelial cells that cover the interior surface of blood vessels. It is not only the barrier located between circulating blood and tissues but also a vital organ involved in numerous functions. Under physiological conditions, the endothelium maintains cardiovascular homeostasis via a wide range of biologically active substances such as cytokines, chemokines, and growth factors [1]. It is mainly involved in the regulation of vascular tone, fluid homeostasis, and host defense [2]. Unfortunately, chronic inflammation, oxidative stress, and shear stress cause disorders of endothelium function that induce procoagulant properties, leading to severe sepsis. They are also responsible for sickle cell disease, macular degeneration, prematurity, or diabetic retinopathy [3]. Endothelial dysfunction leads to atherosclerotic lesions and, consequently, an increased risk of cardiovascular events such as idiopathic pulmonary arterial hypertension, stroke, infarction, or heart failure [4]. There is a growing amount of evidence that endothelial cells can serve as sources of myofibroblasts in fibrosis, such as cystic, kidney, heart, dermal, pulmonary, and intestinal fibrosis, as well as cancer-associated fibroblasts (CAFs) in neoplasia [5,6,7,8,9,10,11,12,13,14,15].

It has been estimated that around 45% of natural deaths yearly can be associated with different fibrotic disorders in the USA [16]. Organ fibrosis is a common pathological state of slightly known etiology, resulting in chronic tissue injury defined as an increasing production and deposition of extracellular matrix (ECM) components [17]. Chronic inflammation causes fibrosis tissue to recruit numerous activated fibroblasts called myofibroblasts. The myofibroblast was initially described in the granulation tissue of wound healing as the cells with prominent cytoplasmic microfilament bundles and peripheral focal adhesions. Electron microscopy revealed that myofibroblasts characterized the abundant expression of α-SMA, the isoform of actin specific for smooth muscle cells [18]. Myofibroblasts are a heterogeneous group of cells with a comprehensive source of cells, including fibroblasts, circulating bone marrow-derived cells, and epithelial or endothelial cells [19]. They are characterized as α-SMA-positive myofibroblasts, which are the principal source of the enormous extracellular matrix (ECM), including collagen type I, fibronectin, hyaluronan, and elastin [19,20,21]. In turn, cancer-associated fibroblasts (CAFs), a type of activated fibroblast located in the cancer niche, are cells with significant heterogeneity and plasticity. CAFs are widely described as the leading promoter of tumor progression and metastasis. It should be mentioned that CAFs may also have certain tumor-suppressive functions in the early stage of tumors [22].

**Figure 1:**
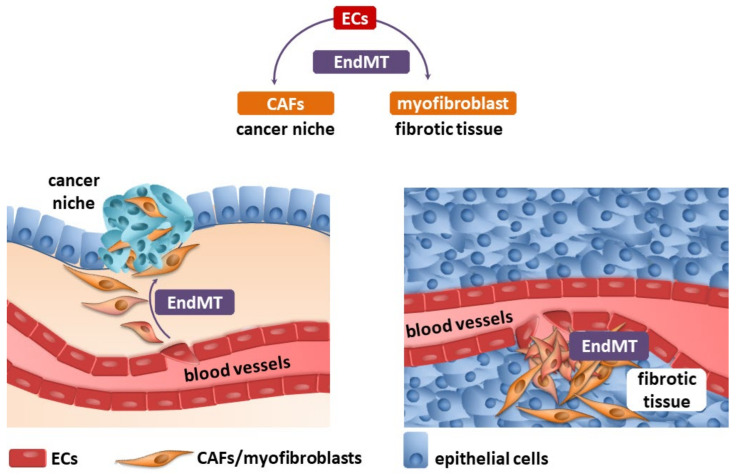
Endothelial cells (ECs) undergo an endothelial–mesenchymal transition (EndMT), which are the source of cancer-associated fibroblasts (CAFs) and myofibroblasts (detailed description in the text).

Fibrosis and neoplastic disease are two distinct and distant disease entities. However, they have a common denominator: the excessive deposition of ECM proteins by myofibroblasts/CAFs, which changes the homeostasis within the niche. One of the well-known sources of myofibroblasts/CAFs is endothelial cells transdifferentiated into them by the endothelial–mesenchymal transition (Figure 1).

## 2. Endothelial–Mesenchymal Transition

**Figure 2:**
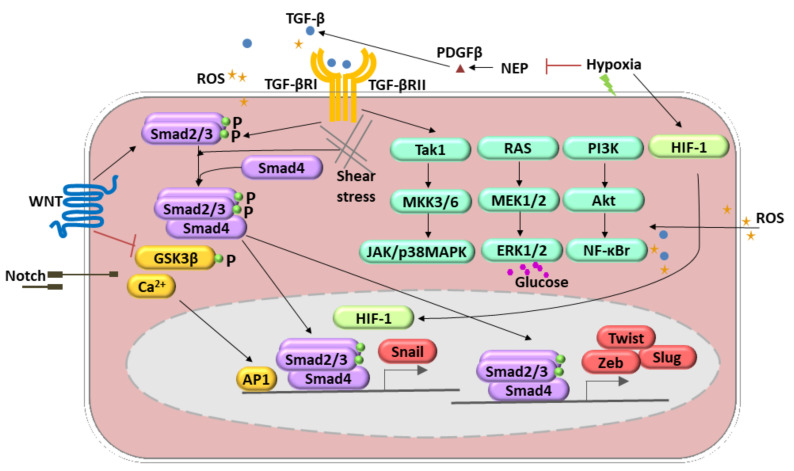
Molecular pathways involved in the endothelial-to-mesenchymal transition (EndMT) regulation. These include transforming growth factor (TGF)-β, TNF-α, BMP, FGF, IL-1 β, Notch, WNT signaling, oxidative stress, and shear stress. TGF-β-induced EndMT involves the canonical Smad2/3 pathway. Additionally, it can activate Smad-independent pathways (1. Tak1, MKK3/6, JAK/p38MAPK; 2. RAS, MEK1/2, ERK1/2; 3. PI3K, Akt, NF-κB). Hypoxia induces EndMT through the effects of HIF-1α activation of Snail1. Shear stress forces (represented by undulating arrows) induce EndMT through several different molecular mechanisms. Other mechanisms include reactive oxygen species (ROS) generation and activation of NF-κB followed by the PI3K, resulting in the increased production and accumulation of ROS (yellow stars). A high level of glucose acts as an ERK1/2 activator and, therefore, regulates the EndMT.

The endothelial–mesenchymal transition (EndMT) was initially observed during heart development [23,24]. Several inflammatory mediators, including pro-inflammatory cytokines (e.g., interleukin 1-β, IL 1-β; tumor necrosis factor-α, TNF-α), growth factors (e.g., fetal grown factor, FGF), oxidative stress, shear stress, and toxins, induce the conversion of endothelial cells into mesenchymal fibroblast-like cells that promote disease progression [25,26] (Figure 2, Table 1). EndMT appears to be regulated by complex molecular mechanisms and different signaling pathways. Members of the Tumor Growth Factor-β (TGF-β) family are the most known inducers of EndMT. Three TGF-β members, TGF-β1, TGF-β2, and TGF-β3, act through TGF-β type I and II receptors to form multimeric complexes and subsequently launch the downstream Smad-dependent and Smad-independent signaling pathways [19]. The Smad-dependent signaling pathway is essential for increasing the expression of cell-adhesion-suppressing zinc-finger transcription factors (TF) such as Snail, Twist, Zeb, and Slug [27,28]. In the canonical pathway, induction of TGF-β receptors causes activation of the TGF-β type I receptor that binds and phosphorylates Smad2/3, which interacts with Smad4 to form a transcription complex that translocates to the nucleus and triggers the expression of the genes mentioned above [19]. In addition, certain TGF-β family members (TGF-β2, BMP2, and BMP4) induce EndMT by signaling through the TGF-β type II receptor (TGFBR2) [19]. The in vivo relevance of this mechanism is illustrated by the EC-derived heterotopic ossification observed in patients with fibrodysplasia ossificans progressiva, which is due to an overactive mutant TGFBR2 [29]. The crucial role of the TGF-β super-family in the EndMT induction has been validated in cell line studies and in vivo mice experiments. The knockdown and knockout of several TGF-β signaling-related genes, such as Smad2, Smad3, and TGFBR2, prevented EndMT [29].

Several other signaling pathways are involved in the induction of the EndMT. The Wnt signal transduction is run via Smad-dependent TGF-β signaling, and canonical (i.e., involving β-catenin) and noncanonical Wnt signaling pathways [30], whereas Notch pathway activation resulted in Snail upregulation [31]. Additionally, it has been shown that Kaposi’s sarcoma-associated herpesvirus causes EndMT via Notch signaling independently of the TGF-β pathway [32]. Within noncanonical pathways, Rho-GTPase-actin and Smad-independent signaling pathways such as Akt/NF-ĸB and MAPK/ERK can be recognized [33,34,35,36] (Figure 2). Recent evidence also suggests that small RNAs, particularly microRNAs (miRNAs) and long noncoding RNAs (lncRNAs), are crucial mediators of EndMT [37]. Hypoxia is the condition able to induce the EndMT. These molecular pathways engage Snail and hypoxia-inducible factor-1 α (HIF-1α) upregulation. Those processes were observed in radiation-induced pulmonary fibrosis [7]. HIF-1 may also decrease neprilysin (NEP) and, in this way, upregulated platelet-derived growth factor (PDGF)-β) and stimulation of TGF-β1 signaling [38]. Reactive oxygen species (ROS) works by differential pathways. They are able to induce TGF-β expression and, therefore, via a positive feedback loop, lead to ROS production. Secondly, ROS might act as activators of nuclear factor-κΒ (NF-κB) signaling that stimulate EndMT synergistically with TGF-β [39]. Moreover, one of the crucial enzymes responsible for ROS production, NADPH oxidase 4 (NOX4), mediated the TGF-β-dependent production of myofibroblasts by EndMT [40].

**Table 1:** Molecular pathways involved in EndMT.

EndMT Inductor	Receptors	Molecular Pathways	Refereces
TGF-β1, TGF-β2, TGF-β3	TGFBR1, TGFBR2	Smad-dependent pathways (Smad2, Smad3, and Smad4)	[27,28]
TGF-β1, TGF-β2, TGF-β3	TGFBR1, TGFBR2	Smad-independent pathways include the mitogen-activated protein kinase (MAPK) family of serine/threonine-specific protein kinases, phosphatidylinositol 3-kinase (PI3K), RhoA, Rac, c-Abl, and protein kinase C (PKC)-δ. MAPK pathways: extracellular signal-regulated kinase (ERK), p38 MAPK, and c-Jun NH2-terminal kinases (JNK).	[33,34,35,36]
Notch	NOTCHR	Notch and TGF-β synergistically stimulate Snail expression	[31]
Notch	NOTCHIR	GSKβ inhibiton, calcium ions upregulation	[33]
Wnt	Frizzed	Smad-dependent pathways (Smad2, Smad3, and Smad4)	[30]
Wnt	Frizzed	GSKβ inhibiton	[30]
HIF-1		Neprilysin downregulation induces upregulation of PDGF-β and finally TGF-β1 signaling induction (hypoxia)	[38]
ROS	TGFBR1, TGFBR2	TGF-β expression resulted in ROS production in the positive loop	[39]
ROS		Induction of NF-κB signaling that, with TGF-β pathway, stimulated EndmT	[39]
NOX4		ROS production caused TGF-β pathway induction	[40]
Shear stress		High shear stress via ERK5 inhibits EndMT	[41]
Shear stress		Cyclic strain, caused by a perpendicular stretching force on the vessel wall, has been shown to potentiate EndMT by augmenting both TGF-β and Wnt signaling	[42,43]
High glucose		ERK1/2 phosphorylation	[44]

Shear stress that ensures homeostasis of ECs can modulate the EndMT via TGF-β-dependent signaling independent of its intensity. The in vivo and organ-in-chip experiments have shown that high shear stress appears to inhibit EndMT [41] via extracellular-signal-regulated kinase 5 (ERK5), whereas ERK5 overactivation prevents the EndMT in cells exposed to a disturbed flow or are stimulated by TGF-β under static conditions [45]. Different mechanical stresses, termed cyclic strain, and caused by a perpendicular stretching force on the vessel wall, have been shown to potentiate the EndMT by augmenting both TGF-β and Wnt signaling [42,43]. High glucose concentrations have been shown to cause EndMT, involving extracellular signal-regulated kinase (ERK) 1/2 phosphorylation.

**Figure 3:**
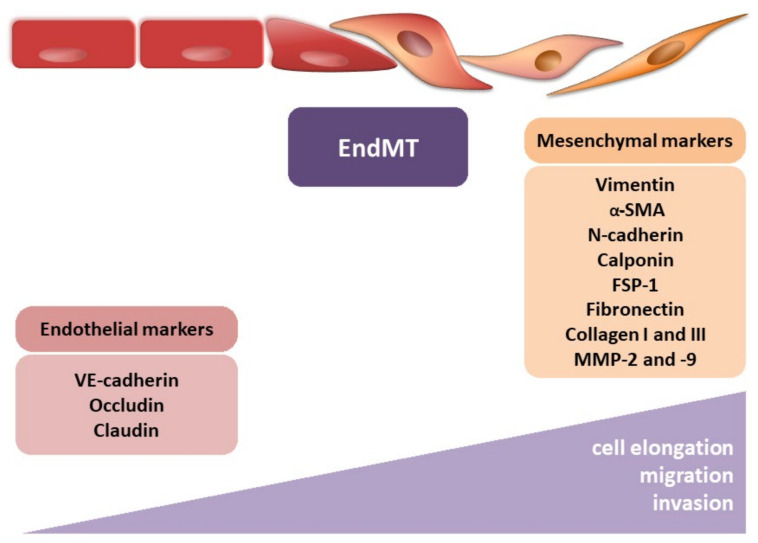
Endothelial–mesenchymal transition. The figure illustrates the morphological modulation (cell elongation) and increasing migration and invasion ability of mesenchymal cells. The observed phenotypic alterations are accompanied by changes in gene expression, i.e., decrease in endothelial markers (VE-cadherin, occludin, and claudin) and increase in mesenchymal markers (vimentin, α-smooth muscle actin (α-SMA), N-cadherin, calponin, fibroblast-specific protein-1 (FSP-1), fibronectin, collagen I, collagen III, metalloproteinase-2 (MMP-2), and metalloproteinase-9 (MMP-9)).

During EndMT, endothelial cells lose their typical phenotype and acquire mesenchymal features, characterized by a shift in endothelial markers toward mesenchymal ones (Figure 3). However, identifying CAFs, it should be remembered that despite many CAF biomarkers, none of them are specific to CAFs [44]. Their expression depends on the cancer type and probably the organ undergoing fibrosis. The endothelial markers commonly include PECAM (CD31), von Willebrand factor (vWF), VE-cadherin (CD144), tyrosine kinase with immunoglobulin-like and EGF-like domains 1 and 2 (TIE1 and TIE2), eNOS, and platelet-derived growth factor (PDGF). To confirm the occurrence of the EndMT, the most often detected mesenchymal markers include α-smooth muscle actin (α-SMA), N-cadherin, calponin, fibroblast-specific protein-1 (FSP-1), vimentin, fibronectin (FN), collagen types I and III, and matrix metalloproteinase 2 and 9 (MMP-2 and MMP-9, respectively). It should be mentioned that other frequently used typical EMT markers, such as E-cadherin, claudin, occludin, and cytokeratin, are not usually used in studies of EndMT [46,47].

**Figure 4:**
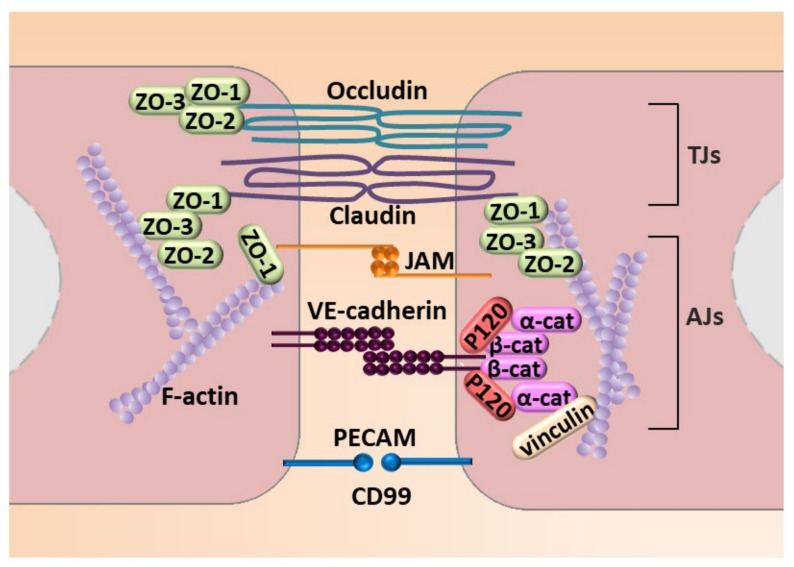
Molecular mechanisms engaged in endothelial cell–cell junction. The figure illustrates adhesion molecules that regulate AJs that consist of VE-cadherin with zonula occludes-1, -2, and -3 (ZO-1, ZO-2, and ZO-3) that interact with a complex of P-120, and a α-cathenin and β-cathenin (α-cat and β-cat, respectively) complex that connects VE-cadherin to stress fibers created by fibrillar actin (F-actin). TJs are created by occludin and claudin and JAM that interacts with ZO-1, ZO-2, and ZO-3. An additional element of cell–cell contact is the PECAM.

Next to molecular modulation, EndMT is manifested by profound morphological and functional changes. ECs that undergo EndMT are characterized by a phenotypic switch involving a loss of cellular adhesion (Figure 4) due to the downregulation of proteins involved in cell–cell junctions (adherens junction (AJs) and tight junctions (TJs)) and cytoskeletal reorganization, which converts tightly compacted cobblestone-like cells into spindle-shaped cells with no apical–basal polarity. EndMT-derived cells thus exhibit an enhanced migratory potential and increased extracellular matrix production, both of which are hallmarks of invasive cells [48] (Figure 4).

Such a reorganization of the cytoskeleton converts EC’s apicobasal polarity toward a front-end back polarity to form spindle-shaped cells with enhanced properties of migration [48] (Figure 3). As a consequence of the processes mentioned above and the influence of the effectors, cell–cell connections are disrupted, and the pro-migratory, pro-inflammatory, and pro-secretory abilities of the cells are increased. That chronic state contributes to the development of fibrotic and neoplastic diseases.

## 3. Cytoskeleton in Endothelial–Mesenchymal Transition

A cellular cytoskeleton is a network of fibrous protein structures forming a scaffold for cell organelles. It plays an essential role in maintaining cell shape, membrane dynamics, intracellular transport, organelle positioning, cell polarization, movement, and division. Three types of structures constitute the basis of the cytoskeleton: intermediate filaments, microtubules (MT), and actin cytoskeleton (microfilaments) [49]. Together with associated proteins regulating their polymerization, motor proteins, and proteins connecting individual elements of the cytoskeleton, these filaments form a functional structure.

The first type of biopolymer is intermediate filaments responsible for ensuring the stability of the cell structure. The primary role of the second type of filament, microtubules, is to counteract mechanical stresses, transport intracellular organelles, as well as build the karyokinetic spindle. The third type of biopolymer is the actin cytoskeleton (microfilaments), which is mainly responsible for cell movement but also cell adhesion and migration [49].

The interrelationship between the cytoskeleton elements and participation in the transmission of environmental stimuli make the cytoskeleton play a key role in the proper development and functioning of tissues and organs, and thus in maintaining homeostasis of the organism [50]. The cytoskeleton is a dynamic system in which both the exchange of subunits in the already existing polymers and the local or global reconstruction of the fiber network take place (possible through the processes of polymerization and depolymerization of individual filaments) [51]. Such cytoskeleton dynamics enables the elimination of damaged subunits or fragments of structures and their replacement by proper units and the reorganization of the cytoskeleton in response to various internal and external stimuli, e.g., during cell division or differentiation and cell migration. Disorders in the proper functioning of the cytoskeleton lead to many serious diseases (e.g., myopathy, neuropathy, ciliopathy, and neoplastic diseases) [52].

### 3.1. Microfilaments

Actin is one of the most abundantly expressed proteins in eukaryotic cells. Its monomers, known as globular actin (G-actin), possess polymerization capability and form fibrillar actin (F-actin) microfilaments. The actin network undergoes continuous directional polymerization and disassembly, dependent on an actual equilibrium between G-actin and F-actin [53]. The actin filaments interact with numerous binding and contractile proteins and therefore provide the modulation source of cell morphology and migration ability [54,55].

As described above, EndMT is characterized by cell shape transformation from cobblestone-like to strongly elongated spindle-like. Those modulations are mainly dependent on actin microfilament rearrangement [56]. Endothelial cells are organized in a compact monolayer, and tight and adherens junctions ensure its integrity. Claudins, occludin, and zonula occludens proteins ZO-1, ZO-2, and ZO-3 are the main elements of tight junctions. Two adjacent endothelial cells are connected by extracellular domains of VE-cadherin, whereas its intracellular fragment binds to catenins and other proteins that link VE-cadherin to microfilament fibers (Figure 4). During EndMT, the tight junctions are degraded. First, VE-cadherin is phosphorylated on the tyrosine residue, which consists of the signal for subsequent internalization, and is cleaved. Simultaneously, phosphorylation of VE-cadherin causes activation of small G-protein, Rac1, and its localization of the leading edge. As a result, cells acquire migratory features [57].

The increased cell movement is associated with actin cytoskeleton remodeling and focal adhesion organization. In more detail, they were observed in myofibroblasts’ stress fibers containing mainly α-SMA organized in parallel microfilaments. Those structures are periodically cross-linked with actin-associated proteins such as α-actinin and myosin II. The stress fibers are linked to the ECM via focal adhesion, the dynamic protein complex that allows the cells to interact with environments. Integrins, focal adhesion kinase (FAK), integrin-linked kinase (ILK), filamin, tensin, vinculin, paxillin, and talin have been described among the focal adhesion proteins [58].

As mentioned above, EndMT forces cytoskeletal remodeling dependent on the Rho GTPase family (resulting from activation of Smad-dependent and Smad-independent pathways). One of the main consequences of EndMT induction and cell morphology remodeling is the recruitment of α-SMA into the fibrillary structures of F-actin, which are essential to forming the stress fibers located along the long axis of the myofibroblast.

**Figure 5:**
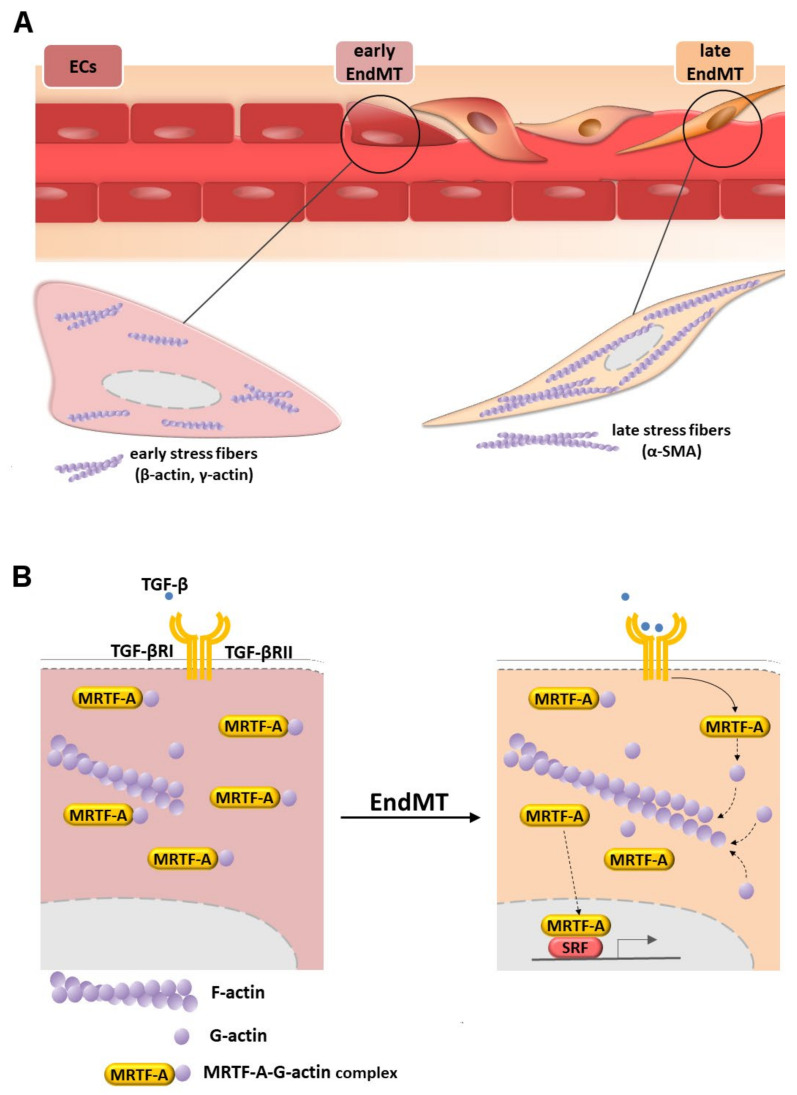
Microfilament remodeling during EndMT-induced stress fiber formation. (**A**) The early stages of EndMT are accompanied by early, non-maturated stress fibers composed of β-actin and γ-actin. In contrast, late stages of EndMT are characterized by late stress fibers created by α-SMA-enriched microfilaments. (**B**) Decomposition of actin microfilaments requires the recruiting of G-actin, which is released from the G-actin-MRTF-A complex. As a result, free MRTF-A translocates to the nucleus and induces the transcription of protein involved in the regulation of the cytoskeleton reorganization (α-SMA, focal adhesion kinase (FAK), and integrin-liked kinase (ILK)) and cell contraction (caldesmon and tropomyosin).

In nonmuscle cells, two actin isoforms, β and γ, were discovered that characterized different cellular distributions [57]. β-actin is located in bundles, ventral stress fibers, intercellular junctions, and contractile mitotic rings, and it regulates cell connection and contraction. γ-Actin is mainly found in dorsal stress fibers in non-migrated cells and in lamellar, cortical, and lamellipodia structures in motile cells. In cardiac fibroblasts in the intermediate stages of myofibroblast transdifferentiation, protomyofibroblasts expressed β-actin and γ-actin located in early stress fibers [56,59,60] (Figure 5).

In contrast, fully differentiated myofibroblasts are characterized by the abundant expression of α-smooth muscle actin (α-SMA) that creates the microfilament bundles and stress fibers and organizes fibronexus adhesion complexes [61] that are responsible for interaction with the ECM. As a result, myofibroblasts maintain the cellular contractile force through the network of cytoskeletal proteins (Figure 5). They secrete mainly collagen I, elastin (ELN), fibronectin (FN1), and tenascin (TNC) that displace other ECM proteins. Additionally, myofibroblasts/CAFs secrete numerous matrix metalloproteinases (MMPs) that facilitate cell migration (tumor cells in cancer and myofibroblasts in fibrosis) through the ECM and basement membrane (BM) [62].

Myocardin-related transcription factors (MRTFs) are expressed in numerous human tissues in two isoforms, MRTF-A and MRTF-B. They act as the transcriptional modulators in the physiological and pathological conditions such as cancer and fibrosis [63,64]. Under physiological conditions, monomeric G-actin is complexed with MRTF-A or MRTF-B, which sequesters MRTFs in the cytoplasm. After cell stimulation, e.g., EndMT, as a result of Rho-GTPase activation (noncanonical pathways), MRTF-A-G-actin or MRTF-B-G-actin complexes dissociate and release MRTFs. Free MRTFs translocate to the nucleus, where they act as the co-activators of serum response factor (SFR) via the conserved CArG box DNA element [65,66,67] or as the direct transcription factor [62].

MRTFs were first described in Schremberg and co-workers’ studies where the authors revealed that MRTF-A is involved in cellular transformation into myofibroblasts via Smad-dependent pathways [68]. A later study showed that MRTFs induce the expression of zinc-finger transcription factors. MRTF-A induces a TWIST1 upregulation in a STAT3-dependent manner [69], whereas both MRTFs isoforms are the direct inductors of Snail expression [70].

MRTF-A is also associated with activation of the transcription of numerous genes during EndMT such as fibronectin, vinculin, talin, focal adhesion kinase (FAK), integrin-linked kinase (ILK), and contraction protein: SM22α, α-SMA, caldesmon, and tropomyosin [65,66,67]. We also demonstrated that during EndMT in HMEC-1 cells, the upregulation of integrin-linked kinase (ILK) enhanced MRTF activation via RhoA and Rac-1-MMP9 via inside-out integrin activation. We underlined the role of the ILK-MMP9-MRTF axis as critical for EndMT [67].

### 3.2. Microtubules

In the interphase, the heterodimer of tubulin-α and tubulin-β binds to the minus terminus to one of the tubulins-γ located in the rings in the microtubule-organizing center (MTOC). It is the first step of microtubules polymerization. Adding the next heterodimers to the plus end of the previous one dimer causes the growth of microtubules, the unbranched, cylindrical fibers [71].

The rate of polymerization and depolymerization of microtubules depends on three factors: the isotype of tubulin-α and -β, post-translational modification within individual subunits, and the profile of microtubules-associated proteins (MAPs). Among tubulins-α and -β, a number of subunits are distinguished (eight tubulin-α and seven tubulin-β were revealed in mammalian cells so far) [71].

The regulation of the level of particular tubulin subunits, essential for the functioning of microtubules, is possible due to the diversity of the 3′-UTR region through the regulation of mRNA stability [72]. The individual β class subunits differ only in the sequence of 15 amino acids located at the carboxy terminus of the molecule, which enables specific interactions of microtubules with MAPs [73]. It is also a place of post-translational modifications, providing functional specificity of the different isotypes. The changes described so far within the β class tubulin include acetylation, polyamidation, phosphorylation, glycation, and glutamylation [30].

**Figure 6:**
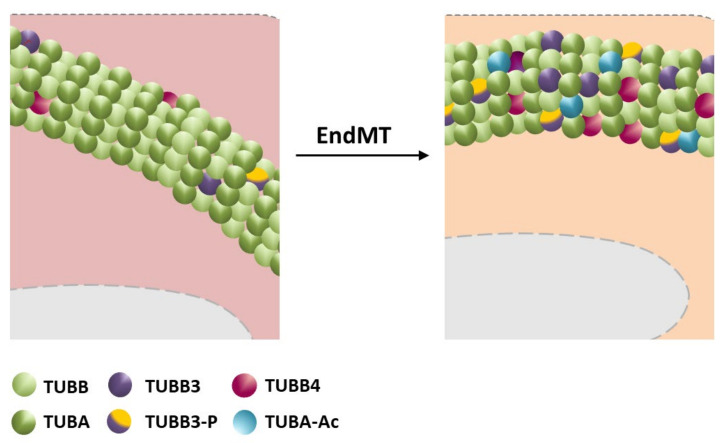
Microtubules reorganization during EndMT. EndMT is accompanied by the upregulation of β3-tubulin (TUBB3) and β4-tubulin TUBB4, as well as increased phosphorylation of β3-tubulin and acetylation of α-tubulin subunits (detailed description in manuscript).

Analysis of the EndMT cellular models of fibrosis induced by TGF-β1 treatment or transfection with Snail factor revealed the modulation of tubulin-β3 and tubulin-β4 ex-pression [74] (Figure 6). Initially, this subunit was considered a marker of resistance to the taxanes or vinca alkaloids used in treating ovarian, lung, stomach, pancreatic, and breast cancer [75,76,77,78,79]. The role of tubulin-β3 in regulating microtubule polymerization (significantly increasing) also indicates its significant role in transforming the cells toward the mesenchymal profile [80,81]. The silencing of tubulin-β3 caused a reduction in migration ability, with the differences particularly evident in the cohort migration (wound healing) [19,74]. Similar results were observed in the modulation of tubulin-β4 expression, which confirmed that both isoforms are involved in the regulation of mesenchymal cell movement.

Cell adhesion and migration alteration are also possible due to microtubule polymerization dynamics, mainly dependent on β-class isotypes composition [82,83,84,85,86]. Microtubules enriched in tubulin-β3 subunits affect the direction of cell migration, but not their rate during EndMT [87,88,89]. It has also been shown that microtubules containing more tubulin-β3 are less stable than those composed of multiple tubulin-β2 or tubulin-β4 subunits [90] as a result of faster GTP hydrolysis within tubulin-β3 than in other β-tubulin subunits. This results in the intensification of microtubule depolymerization and an increase in their susceptibility to a mitotic catastrophe [89].

It has been confirmed that post-translational modifications (PTMs) of tubulins influence the regulation of the rate of microtubule polymerization, and thus their functions. They concern the carboxy terminus of the subunits and are usually associated with regulating interactions with microtubule-associated proteins (MAPs). The influence of post-translational modifications on the development of diseases also caused by EndMT is not yet known. Nevertheless, it is known that changes in the level of tubulin phosphorylation regulate cell adhesion and migration [91,92,93]. It has been shown that TGF-β2 strongly stimulates the modification in the endothelial cells in later EndMT stages [73]. Phosphorylation probably on Ser-172 is regulated by phosphatidylinositol 3-kinase [73] (Figure 6). Numerous studies have demonstrated the crucial role of tubulin phosphorylation in their interaction with microtubule-associated proteins. This correlation was observed in MAP4, TOG (tumor-overexpressed gene), and stathmin. By binding through phosphorylated serine or threonine residues, these proteins, contained in the tubulin structure, modulate microtubule polymerization/depolymerization [93,94,95,96,97].

The knowledge about tubulin’s role in EndMT is strongly limited. The shear stress induced by mechanical ventilation resulted in DRD1 downregulation in surgical patients and mice. It has been revealed that cyclic stretch-induced glycogen-synthase-kinase-3β activation led to phosphorylation and activation of histone deacetylase 6 (HDAC6), which resulted in the deacetylation process of α-tubulin, whereas dopamine attenuated the mechanical stretch-induced deacylation of α-tubulin and subsequent endothelial hyperpermeability through DRD1 signaling. Upon activation, DRD1 signaling attenuated mechanical stretch-induced α-tubulin deacetylation and subsequent lung endothelial barrier dysfunction through the cAMP/exchange protein activated by cAMP (EPAC)-mediated inactivation of HDAC6 [98] (Figure 6).

Recently, it has been shown that primary cilia, microtubule-based organelles found in most mammalian cells, are engaged in EndMT. The primary function of those structures is the transmission of microenvironmental clues into intracellular signals for molecular and cellular responses. Primary cilia are essential for proper vascular development and maintenance of structural integrity through calcium and nitric oxide signaling. In adult mice, primary cilia are particularly abundant in ECs of arterial regions experiencing low shear stress, a condition that is typically observed in wide-neck intracranial aneurysms. In the absence of primary cilia, ZO-1 expression levels are reduced, disorganizing intercellular junctions resulting in increased endothelial permeability [99].

### 3.3. Intermediate Filaments

Intermediate filaments (IFs), in contrast to microtubules and microfilaments, contain numerous proteins that characterize tissue specifically [100,101]. They are located in the plasma membrane, maintain cell shape and traction forces between cells, and protect cells from disruption. IFs were described as the most flexible and insoluble structures in the cells [102]. Despite different structures, they are organized with similar structural domains [103].

More than 70 proteins that belong to IFs are divided into six types regarding their amino acid sequences. Types I and II consist of two groups of keratins (15 different proteins in each group), expressed in epithelial cells. Whereas acidic keratins in type I have been collected, the neutral and basic keratins belong to type II. In epithelial cells, at least one type I and one of type II keratins are expressed and form intermediated filaments. The type III intermediate filament proteins include vimentin. It is described in numerous cells, including fibroblasts, smooth muscle cells, white blood cells, and myofibroblasts. Desmin, a protein specifically expressed in muscle cells, also belongs to type III IFs. Type IV consists of three neurofilament (NF) proteins (designated NF-L, NF-M, and NF-H for light, medium, and heavy, respectively) revealed in numerous maturated neurons. They are abundantly expressed in the axons of motor neurons. Another type IV protein (α-internexin) characterized the earlier stage of neuron development prior to the expression of the neurofilament proteins. The single type VI intermediate filament protein (nestin) is expressed even earlier during the development of neurons in stem cells of the central nervous system. Lamins belong to type V IFs located in the nucleus of most eukaryotic cells as the components of the nuclear envelope [103].

The crucial IFs observed in myofibroblasts is vimentin. It is one of the most popular markers studied during EndMT [104]. Vimentin is observed in endothelial cells, but its level and function are different than in a mesenchymal cell. While, in nonmigrated cells, its expression is low, and the presence is associated with cell shape maintenance than in motile mesenchymal cells, vimentin is abundantly expressed and is involved in the highly dynamic cytoskeleton remodeling [105]. It has been observed that the silencing of vimentin expression was associated with the inhibition of migration properties in the myofibroblasts [106].

As silencing vimentin would switch mesenchymal cells into the epithelial phenotype, vimentin’s overexpression would change epithelial cells into the mesenchymal phenotype. Therefore, the level of vimentin expression seems to be strongly linked to the cells that manifested the mesenchymal phenotype. The level of vimentin expression was significantly upregulated in clinical renal cell carcinoma specimens compared to normal tissues by immunohistochemistry assay. Vimentin is regulated by miR-138 and miR-141, which participate in cell migration, adhesion, and signaling processes [107]. It stabilized focal adhesion and therefore regulated cell migration. It is a signal transducer from the ECM to the nuclei [108,109].

Vimentin is also involved in cell-matrix adhesions and strengthens the adhesion sites [110,111,112]. For instance, vimentin regulates the specificity of focal adhesion–extracellular matrix (ECM) interactions through vimentin-associated matrix adhesions (VMAs), which assemble in actively migrating cells, interacting with actin microfilaments through vinculin and with vimentin through vinculin and with vimentin IFs through plectin [113]. It has been revealed that vimentin interacts with contractile actomyosin arcs, consequently regulating the localization of arcs and morphogenesis of flat lamellae in migrating cells [114]. Emerging evidence suggests that vimentin is involved in cytoskeleton-regulated mechanosensing, a fundamental feature for controlled cell motility [115].

Moreover, vimentin has numerous phosphorylation sites critical for the architecture of the cellular filaments. The vimentin network is involved in phosphorylation and dephosphorylation, enabling integrin-mediated cell adhesion and facilitating directional cell motility [116,117]. In keeping with such roles, identifying the coordinated proteins and molecules modulated by vimentin might reveal the IF-mediated cytoskeleton cross-talk associated with liver fibrosis.

## 4. EndMT Inhibition in Fibrosis and Cancer Treatment

As has already been discussed, EndMT is instrumental in the pathogenesis, development, and progression of various human pathologies, including multiple fibrosis and tumors. Due to the above fact, there is great interest in the abolition or modulation of EndMT as a new therapeutic approach for treating these disorders. Indeed, intensive research is currently being carried out focusing on the identification of new compounds, natural substances, and pharmacological agents intended to act as EndMT inhibitors and their use as a potential therapeutic agent in the treatment of various diseases in which EndMT is shown or suggested to play a role in their pathogenesis.

Targets postulated for these inhibitory effects are quite diverse. Still, a reasonably large group includes compounds with the ability to modulate the reorganization of the cytoskeleton as an essential cellular component directly responsible for the implementation of the EndMT process.

### 4.1. Tubulin Inhibitors

Tubulin inhibitors are the largest group of compounds interacting with the cytoskeleton primarily due to their cytotoxic activity and impressive success in clinical oncology. The main feature that separates them from other anticancer drugs is the mode of action that targets the mitotic spindle, not DNA. Based on the mechanism or site of action, tubulin inhibitors have been classified into tubulin polymerization and depolymerization inhibitors [118].

#### 4.1.1. Tubulin Polymerization Inhibitors

The largest class of tubulin polymerization inhibitors are vinca alkaloids, such as vincristine and vinblastine, known as useful antimitotic anticancer agents acting by preventing MT assembly [119].

Vincristine is applied in leukemia, lymphomas, sarcomas, brain tumors, lung cancer [119], and colon cancer therapy. Vincristine works by decreasing the polymerization rate and arresting cells in the metaphase [120]. This vinca alkaloid is the commonly recommended therapy in patients diagnosed with invasive stages of tumor development [121]. Research on colon cancer cell lines and human material indicates that the tumor gradually becomes resistant to vincristine [122,123]. However, some studies have revealed that vincristine might promote cancer metastasis by CAFs formation [124] and enhance metastatic niche formation [125]. We observed that vincristine therapy might accelerate tumor growth, leading to invasive stages by increasing the number of CAFs in the tumor microenvironment, even in the early stages of tumor development. The described process is regulated by IL-6, TGF-β1, and TGF-β2 secreted by vincristine-treated CAFs [118]. On the other hand, there are also studies showing the involvement of vinca alkaloids in modulating fibrosis. It has been proposed recently that vinpocetine, a synthetic derivative of vincamine (vinca alkaloid), attenuates liver fibrosis [126,127] and cardiac hypertrophy and fibrosis [128].

Recently, nonsteroidal anti-inflammatory drugs (NSAIDs) have been used more frequently to treat invasive cancer. Their high effectiveness was noted in the case of numerous solid tumors. It is believed that the effect of using NSAIDs may be associated with a reduction in inflammation induced by stromal cells [88,129,130]. Moreover, it was noticed that the use of ibuprofen in the treatment of cystic fibrosis, in which multiple fibrosis develops, inhibits their formation by modulating the dynamics of microtubules in epithelial cells [90]. It was investigated whether the application of combination therapy (vincristine with aspirin (ASA) or ibuprofen (IBU)) would overcome the EndMT effect. In those circumstances, vincristine cells cause the release of TGF-β1, TGF-β2, and IL-6, whenever ASA or IBU inhibits those effects. Modulation of the secretion ability seems to depend on the profile of tubulin-β2 and tubulin-β3 in microtubules. As endothelial cells are one of the primary sources of CAFs formation, inhibition of EndMT could prevent the endothelial transition and thus reduce the CAFs population and finally decrease cancer metastasis [124].

Another group of tubulin polymerization inhibitors includes the agents interacting with the so-called colchicine binding site. Colchicine is a well-established alkaloid derived from *Colchicum autumnale*, exhibiting anti-inflammatory and anticancer effects [118]. Additionally, colchicine is also known for its anti-fibrotic properties in various organs, including the lungs [131], kidneys [132], heart [133], and liver [134]. Its mechanisms are based mainly on TGF-β/Smad3 pathway targeting, which leads to attenuation of alcoholic liver fibrosis [135], atrial fibrillation [136], and renal fibrosis [137]. Another very promising agent with anti-fibrotic properties binding to the colchicine site of tubulin is 2-methoxyestradiol, a natural metabolite of the hormone estradiol [138]. It has been revealed that 2-methoxyestradiol attenuated hypoxia-induced fibrosis in systemic scleroderma [139] and radiation-induced pulmonary fibrosis [7,140] through EndMT inhibition.

#### 4.1.2. Tubulin Depolymerization Inhibitors

Taxoids, another group of antimitotic agents, have a mechanism of action based on binding mainly to a domain different from those of vinca alkaloids and colchicine, which inhibit the depolymerization of polymerized tubulin [118]. The most known taxoid drug and probably the most widely used anticancer drug is paclitaxel, isolated from the bark of the *Taxus brevifolia* tree. It has been described before that the TGF-β/Smad pathway is one of the most critical profibrotic routes. Moreover, it has been proposed that binding endogenous Smad proteins to microtubules might negatively modulate TGF-β activity [141]. Thus, it has been suggested that paclitaxel through stabilizing microtubules lead to inhibition of the TGF-β signaling pathway in fibrosis [86]. Indeed, paclitaxel in low doses ameliorates hepatic fibrosis [142], renal fibrosis [143,144], and pulmonary fibrosis [145], and modulates tumor fibrosis in gastric cancer [146] through regulating TGF-β/Smad signaling. On the other hand, it was observed that high-dose paclitaxel might induce scleroderma [147] and pulmonary fibrosis [148] in patients. Thus, although low-dose paclitaxel has a beneficial role in preventing tissue fibrosis, it might be involved in fibrosis induction when a high dose anticancer agent is used.

### 4.2. Vimentin Inhibitors

One of the most known drugs targeting vimentin IFs is Withaferin A (WFA), a steroidal lactone derived from the plant *Withania somnifera* with pleiotropic mechanisms of action concerning its anti-inflammatory, anti-angiogenic, and anti-tumorigenic properties [149,150]. WFA targets and directly binds vimentin and other intermediate filament proteins, and its activity is concentration-dependent. WFA causes vimentin phosphorylation and disassembly in lower doses and downregulates the gene expression in higher doses [151]. It has been shown that WFA prevents renal [152], liver [153], pulmonary [154], corneal [155], and myocardial fibrosis [156]. Resveratrol, another natural compound produced by plants in response to injury or attack by pathogens, suppresses vimentin expression through the TGF-β1 signaling pathway [157]. It was proposed that resveratrol, through vimentin regulation, is implicated in the diabetic retinopathy fibrotic process inhibition [158] and cardiac fibrosis attenuation [157]. Moreover, resveratrol is also able to modulate crosstalk between EndMT and EMT in the tumor microenvironment. It was revealed that resveratrol inhibited the migration ability of breast cancer cells stimulated by HUVEC cells that underwent the EndMT process [159]. Dioscin, a steroidal saponin isolated from medicinal plants such as *Dioscorea nipponica* Makino, is another agent that revealed anti-fibrotic properties through vimentin affecting pulmonary [160] and liver fibrosis [161]. Silibinin was found to inhibit myofibroblast transdifferentiation and reduce fibrosis in a rabbit trabeculectomy model through the effect of the vimentin [162]. Simvastatin treatment that prevented tubular activation and transdifferentiation, processes involved in renal fibrosis, was associated with vimentin downregulation [163]. Similar results for vimentin modulation have also been observed for ursolic acid that inhibits human umbilical vein endothelial cell EndMT and fibrosis [164].

## 5. Conclusions

Consisting of three intracellular filaments, the cytoskeleton plays a fundamental role in maintaining cell shape and regulating migratory properties. Due to cytoskeleton involvement in the EndMT, it is an attractive target for cancer and fibrosis therapy.

The research conducted so far indicates many potential sites of cytoskeleton interaction crucial for the inhibition of endothelium transdifferentiation. However, knowledge about the role of individual cytoskeleton elements in the induction of the mesenchymal nature of endothelial cells and potential inhibitors of these processes is still unsatisfactory, especially compared to a similar EMT process. Due to the high potential of inhibiting the development of two widespread diseases with poor prognoses, further in-depth research into the role of the distribution and interaction of individual filaments during EndMT seems indispensable.
